# Voting Simulation based Agglomerative Hierarchical Method for Network Community Detection

**DOI:** 10.1038/s41598-018-26415-3

**Published:** 2018-05-23

**Authors:** Jianjun Cheng, Xinhong Yin, Qi Li, Haijuan Yang, Longjie Li, Mingwei Leng, Xiaoyun Chen

**Affiliations:** 1 Lanzhou University, School of Information Science and Engineering, Lanzhou, 730000 China; 2 Gansu Resources and Environmental Science Data Engineering Technology Research Center, Lanzhou, 730000 China; 3 Lanzhou Vocational Technical College, Department of Electronic Information Engineering, Lanzhou, 730070 China; 4 Northwest Minzu University, School of Education Science and Technology, Lanzhou, 730030 China

## Abstract

Community detection has been paid much attention in many fields in recent years, and a great deal of community-detection methods have been proposed. But the time consumption of some of them is heavy, limiting them from being applied to large-scale networks. On the contrary, there exist some lower-time-complexity methods. But most of them are non-deterministic, meaning that running the same method many times may yield different results from the same network, which reduces their practical utility greatly in real-world applications. To solve these problems, we propose a community-detection method in this paper, which takes both the quality of the results and the efficiency of the detecting procedure into account. Moreover, it is a deterministic method which can extract definite community structures from networks. The proposed method is inspired by the voting behaviours in election activities in the social society, in which we first simulate the voting procedure on the network. Every vertex votes for the nominated candidates following the proposed voting principles, densely connected groups of vertices can quickly reach a consensus on their candidates. At the end of this procedure, candidates and their own voters form a group of clusters. Then, we take the clusters as initial communities, and agglomerate some of them into larger ones with high efficiency to obtain the resulting community structures. We conducted extensive experiments on some artificial networks and real-world networks, the experimental results show that our proposed method can efficiently extract high-quality community structures from networks, and outperform the comparison algorithms significantly.

## Introduction

Community structure is a significant structural characteristic of many complex networks, in which communities are always closely related to real functional modules in real-world systems, e.g., groups of Web pages^1^ or scientific articles^2^ sharing same topics, pathways in metabolic networks or complexes in protein-protein interaction networks^3–7^, the real social groupings in social networks, such as scientist groups working on specific research fields^8,9^, Jazz musician groups divided according to the locations and race^10^, and affiliations of gang members in the policing area of Hollenbeck, Los Angeles^11^. Therefore, detecting communities from complex networks can help us understand the structural characteristics of networks, and predict further the functional properties according to the structure. Besides this, the existence of community structures in networks can have considerable influences on such dynamic processes as information dispersions^12,13^ and synchronisations^14^ as .l.

Therefore, community detection has received much attention, and a great deal of detection methods have been proposed. For instance, hierarchical methods either repeatedly divide networks into subnetworks^8,9^, or iteratively agglomerate small vertex groups into larger ones^15,16^, or combine both the divisive and agglomerative strategies^17^ to get the resulting community structures. Modularity-optimisation based methods^12,15,16,18,19^ utilise the intention of modularity^9^ – the larger the modularity, the better the corresponding community structure, to extract better results from networks via optimising the modularity as the objective to obtain a higher value. LPA^20^ and variants^21–23^ exploit information-propagation mechanism to detect community structures, they propagate labels in the network, and densely connected vertices in a network can quickly reach a consensus on a unique label, thus form a community. Network dynamics-based methods make use of dynamic processes in networks such as random walk^24–26^, information diffusion^27^, and distance dynamics^28–30^ to explore community structures. Spectral methods engage the eigen-spectra of various of matrices associated with networks, such as the adjacency matrix^31,32^, Laplacian matrix^7,33–35^, transition matrix^11,36,37^, and nonbacktracking matrix^38–40^ to reveal community structures. And density base methods^23,41,42^ use information of shared neighbours between vertices to define the concept of *density* of vertex or partition, then make utilisation of the concept to reveal community structures from networks.

## Motivation

Many of those methods suffer from high-time consumption, so that they cannot be applied to large-scale networks. However, networks originated from real-world systems become larger and larger nowadays, the detecting efficiency is critical for some applications. To be frank, there already exist some methods, e.g., Fast*Q*^15,16^, LPA^20^, and PPC^26^, can detect communities from networks with a relatively lower-time consumption. Fast*Q* identifies communities from the network by repeatedly joining pairs of communities whose merge can lead to the largest modularity increment. The hierarchy corresponding to the largest modularity in the output dendrogram is the resulting community structure. The time complexity of Fast*Q* is *O*(*mh* log *n*), or *O*(*n* log^2^ *n*) for sparse networks, where *m*, *n* are numbers of edges and vertices in the network, respectively; *h* is the height of the dendrogram. For LPA, each vertex in the network is assigned a unique label initially, then each vertex updates its label with the most frequent label in its neighbours. Updating continues until every vertex has a label which occurs most frequently among its neighbours. LPA can obtain the community structures in *O*(*m* + *n*) time consumption, the authors claimed that 95% of vertices or more can be classified correctly by the end of the 5th round of label update, irrespective of size of the network. Owing to its simplicity and efficiency, a series of variants have been derived from LPA. Barber *et al*.^21^ produced a variety of algorithms that propagate labels under constraints. LPAm is one of particular interest, which maximises the modularity corresponding to the final community structure. Chin *et al*.^22^ made use of the number of mutual neighbouring vertices to form the main communities first, then they proposed some independent conditions as constraints of LPA, and utilised the constrained LPA to add the remainder vertices into communities. Finally, they used a vertex-moving strategy to refine the quality of the resulting community structure. Ding *et al*.^23^ introduced a modified label propagation algorithm, DCN, which employs the idea of Fdp algorithm^43^ and Chebyshev inequality to select community centres from the network first, then assigns their labels to their neighbours to form some seed regions, and updates the labels of the other vertices with the ones occurs most frequently in their own neighbours finally. For PPC, it combines the dynamic procedure – random walk in networks, and modularity-optimisation process together to divide networks into sub-networks iteratively. The authors stated that PPC can extract community structures from networks efficiently in approximately linear time complexity.

Nevertheless, these lower-time-complexity algorithms have some deficiencies. For Fast*Q*, it intends to optimise modularity though, its results are always trapped in suboptimal rather than optimal. For LPA series and PPC, almost all of them are non-deterministic algorithms, meaning that running each of them on the same network many times, the uncovered community structures might be different. These shortcomings reduce their practical utilities and limit them from being applied to some real-world applications.

To sum up the above arguments, to extract high-quality and definite community structures quickly from networks is still a challenging problem. To put it in another way, we need community-detection methods do not consider the problem from the single perspective of either detecting efficiency or quality of the results only. To solve this problem, we propose a community detection method that takes both of the two factors into account. The most of significance of the present method is that it is a fast and deterministic method, which can reveal high-quality and definite community structures from networks with a complexity comparable with that of LPA or PPC.

By analysing community structures carefully extracted from many networks, we observed that each vertex and most of its neighbours always belong to the same community, and that vertex and its neighbours in the same community form a small ‘cluster’, each community is composed of several small clusters. In each cluster, there always some vertex who has relative-larger degree than others, the cluster is a group of vertices associated with that vertex with relative-larger degree. This phenomenon is analogous to the voting behaviour in social systems. In the networks abstracted from social systems, there are always some influential individuals in local area, which might be leaders of departments, authorities in some fields, and so forth. If those individuals participate an election which allows nominating freely, every voter will vote for someone having larger influence around himself, with ties broken by selecting the closest one with himself. This procedure will result in many small clusters which is a vertex group surrounding the influential vertex with others.

Motivated by this observation, we propose a method simulating the voting procedure to detect community structures from networks. In the presented method, we make some principles as voting rules, use the degree to reflect the influence of each vertex, and use the similarities between vertices to represent the closeness of them. That is to say, each vertex votes for one of its neighbours whose degree is larger than that of itself. If there are more than one larger-degree vertices having the same influence among its neighbours, the voter vertex votes for the one with the largest similarity with itself. In this way, each vertex votes quickly, and we will obtain many small vertex clusters at the end of voting procedure. According to the above analysis, each community consists of several clusters. Therefore, we take the clusters as initial communities, and merge some of them to construct the resulting community structure finally.

## Results

We have tested the performance of our method on 9 networks, including 2 artificial networks synthesised using LFR benchmark network generator software^44^ and 7 real-world networks. The size of these networks spans from tens to hundreds of thousands of vertices, the statistical information of them are listed in Table 1. These networks can be divided into two categories, one is the two artificial networks and the first three real-world networks in Table 1, in which the ground-truth community structures are already known. The other is the last four ones in Table 1, which are also real-world networks however have no acknowledged ground-truth community structures.

**Table 1:** The statistical information of the networks involved in the experiments.

Network	Vertices	Edges	Communities	Reference
LFR_1000	1000	15135	16	—
LFR_5000	5000	47368	57	—
Dolphin	62	159	4	^45^
Risk map	42	83	6	^48^
Scientists collaboration	118	197	6	^8^
Email	1133	5451	—	^49^
PGP	10680	24316	—	^50^
DBLP	317080	1049866	—	^51^
Amazon	334863	925872	—	^51^

Below, we analyse in detail each of the community structures extracted from the first category of networks individually, and compared the results extracted by our proposal from all 9 networks with those of some popular algorithms.

### Synthetic networks

**Figure 1:**
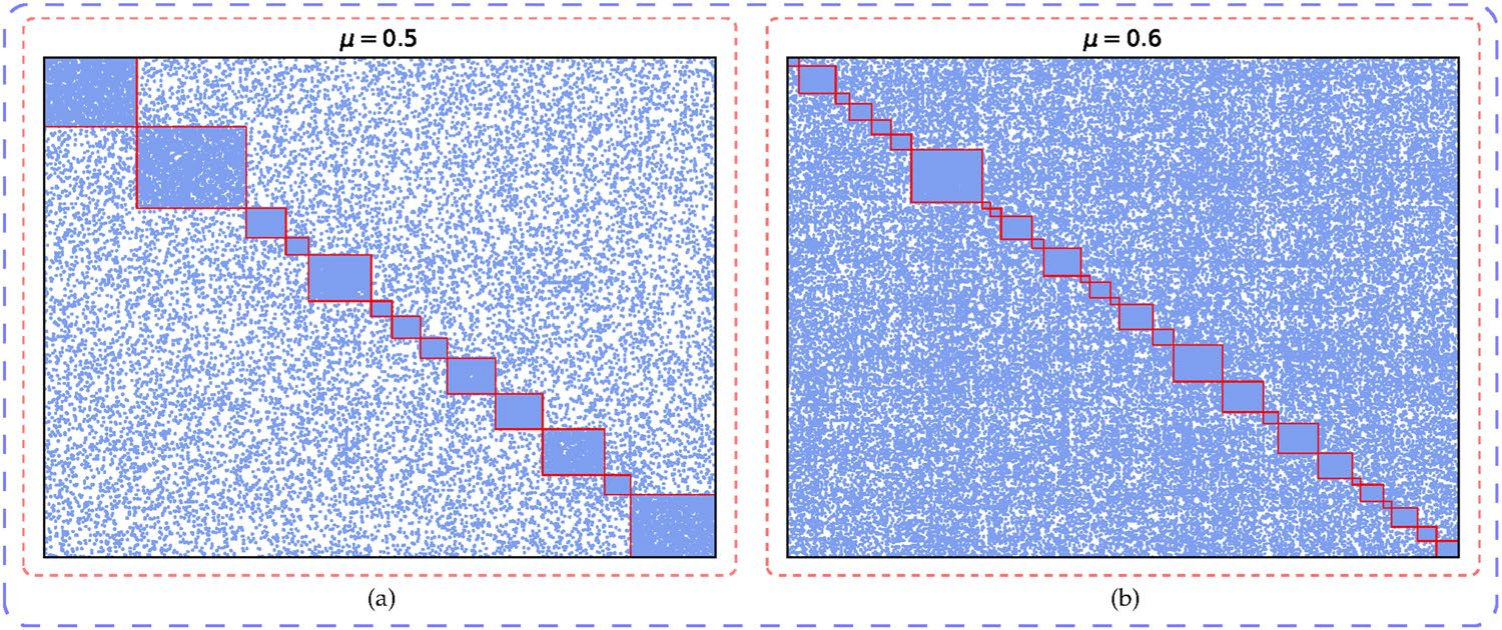
The artificial networks. (**a**) The community structure extracted from the synthetic network containing 1000 vertices, which is generated by setting the key parameter *μ* = 0.5 in the LFR benchmark network generator software. (**a**) The result uncovered from the artificial network containing 5000 vertices, which is synthesised using the same software with *μ* = 0.6.

The two artificial networks are both synthesised using LFR benchmark network generator software^44^, which works with some parameters tuning the properties of generated networks. For the first artificial network, the parameters are set as follows: the vertex number are 1000; the average and maximum degree of vertices are 30 and 50, respectively; both the vertex degree and the community size distributions obey power laws, and the exponent of them are −2 and −1, respectively; the minimum and maximum community sizes are 30 and 100, individually; the mixing parameter *μ* is 0.5. For the second artificial network, it contains 5000 vertices, the average and maximum degree of vertices are 20 and 100, respectively; the power-law-distribution exponents are also −2 and −1, respectively; the minimum and the maximum community contain 50 and 150 vertices, respectively; and *μ* is 0.6. For this software, *μ* is a key parameter, which controls for each vertex the fraction of edges connected to vertices located in other communities. These parameter settings yield the two artificial networks in which the communities are not well-separated one another, so that they can serve well as benchmarks to test the detecting ability of our proposed method. The resulting community structures identified from them by our proposed method are illustrated in Fig. 1, in which each pixel represents an edge, the rectangle areas along the diagonal are corresponding to detected communities. Obviously, the edges inside each of the rectangles are much denser than others, which is consistent with the characteristic of communities.

### Real-world networks

**Figure 2:**
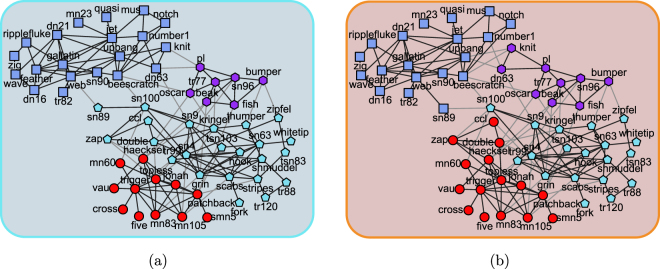
The dolphin social network. (**a**) The ground-truth community structure. (**b**) The community structure detected by our proposed method. The different vertex shapes and colours indicate different communities, the intra-community edges are plotted as black lines, and the inter-community ones are in grey. This illustration style also applies to the next figures.

The dolphins social network was compiled by David Lusseau *et al*.^45^, who observed and studied the behaviour of 62 bottlenose dolphins living in Doubtful Sound, New Zealand for 7 years. The vertices in this network represent the dolphins, and edges associate dolphin pairs being observed co-occurring frequently. The 62 vertices and 159 edges can be partitioned into 4 groups as the ground-truth community structure, as shown in Fig. 2(a). Taken this network as the input, the proposed algorithm extracted the result as shown in Fig. 2(b). Although, some of the vertices are misclassified, the presented method distilled the mainframe of community structure, 4 communities are identified from the network, and the majority of the vertices are classified into the communities correctly.

**Figure 3:**
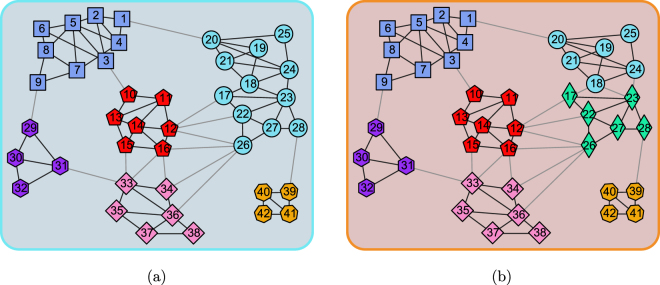
The network corresponding to a map of game Risk. (**a**) The ground-truth community structure. (**b**) The community structure extracted by the proposed method.

The Risk map network is a map loaded in the popular game Risk. It is a map of the Earth, involving 42 territories as vertices, and 83 edges connecting territories which are adjacent. The vertices are naturally partitioned into six communities, corresponding to the six continents in the Earth, which is illustrated in Fig. 3(a). The community structure extracted by the proposed method is as presented in Fig. 3(b). Although, this is a small network, it contains some special vertices, e.g., vertices labelled as ‘12’, ‘16’, ‘26’, the edges associated to them are incident to different communities almost equally, which increases the difficulty of classifying those vertices correctly. Therefore, several community-detection methods can not deal with them well, and tend to introduce misclassification around them^36^. However, our proposed method classified these special vertices correctly, and identified 7 communities from the network. Compared to the ground truth in Fig. 3(a), the two communities located at the right top of the panel in Fig. 3(b) are not merged into one due to the larger value of modularity of the extracted result. This can be verified by Table 2, the modularity of the ground-truth community structure is 0.621, and the modularity corresponding to the extracted result is 0.634, the latter is larger than the former, and the latter is also the largest value among the competitors.

**Table 2:** The experimental results on the first category of networks, the quality of the extracted community structures are measured in terms of modularity (*Q*) and normalised mutual information (NMI).

network	measure	ground truth	Fast*Q*	LPA	LPAm	PPC	Attractor	IsoFdp	proposal
LFR_1000	*Q*	0.43	0.356	0.326	0.385	0.404	0.356	0.36	**0.41**
	NMI	1.00	0.671	0.752	0.89	0.924	0.902	**0.941**	0.925
LFR_5000	*Q*	0.38	0.275	0.122	0.149	0.271	0.197	0.308	**0.342**
	NMI	1.00	0.345	0.304	0.368	0.501	0.536	0.649	**0.776**
Dolphin	*Q*	0.519	0.491	0.503	0.497	0.519	0.495	0.466	**0.522**
	NMI	1.00	0.733	**0.837**	0.744	0.812	0.691	0.629	0.783
Risk map	*Q*	0.621	0.625	0.624	0.567	0.621	0.623	0.519	**0.634**
	NMI	1.00	0.894	0.848	0.888	0.803	0.834	0.714	**0.945**
Scientists collaboration	*Q*	0.739	0.749	0.681	0.587	**0.751**	0.707	0.62	0.739
	NMI	1.00	0.867	0.799	0.704	0.877	0.857	0.775	**0.968**

The largest values are typed in bold.

**Figure 4:**
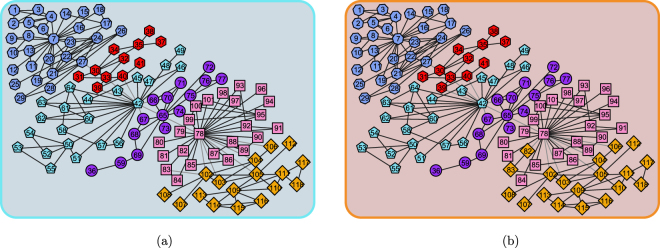
The collaboration network of scientists working at the Santa Fe Institute (Colour on-line). (**a**) The ground-truth community structure. (**b**) The community structure identified by the proposed method.

The scientists collaboration network is the largest component of a network depicted the co-author relationship among scientists working at the Santa Fe Institute, New Mexico, it contains 118 vertices and 197 edges. According to the speciality of the scientists involved, the vertices can be classified into 6 groups. Therefore, this network contains 6 communities naturally, which is shown in Fig. 4(a). The community structure revealed by the proposed method is as exhibited in Fig. 4(b), from which we can see that our proposed method detected community structure from this network with a high degree of success as well, only 2 vertices were misclassified in the incorrect community, it approaches the ground truth mostly.

### Comparison with other methods

To test the performance of our proposed method, we ran it on the 9 networks, and compared the results to those of 6 popular algorithms, namely Fast*Q*^15,16^, LPA^20^, LPAm^21^, PPC^26^, Attractor^28^ and IsoFdp^41^. The first four of them have been discussed previously, which motivated the proposed method to some extent. Attractor is a community detection method utilising distance dynamics. It takes the network as an adaptive dynamical system, in which vertices interact one another. The interaction might make a change on distances among vertices, and the change of distance will affect the interaction in reverse. Such interplay will make vertices belonging to the same community move together step by step, and vertices in different communities depart farther away from each other gradually. IsoFdp is a community detection method based on manifold learning and density-based clustering, which exploits IsoMap^46^ to map the network data into a lower dimensional manifold first, and then extracts communities by clustering the mapped vertices using Fdp algorithm^43^.

**Figure 5:**
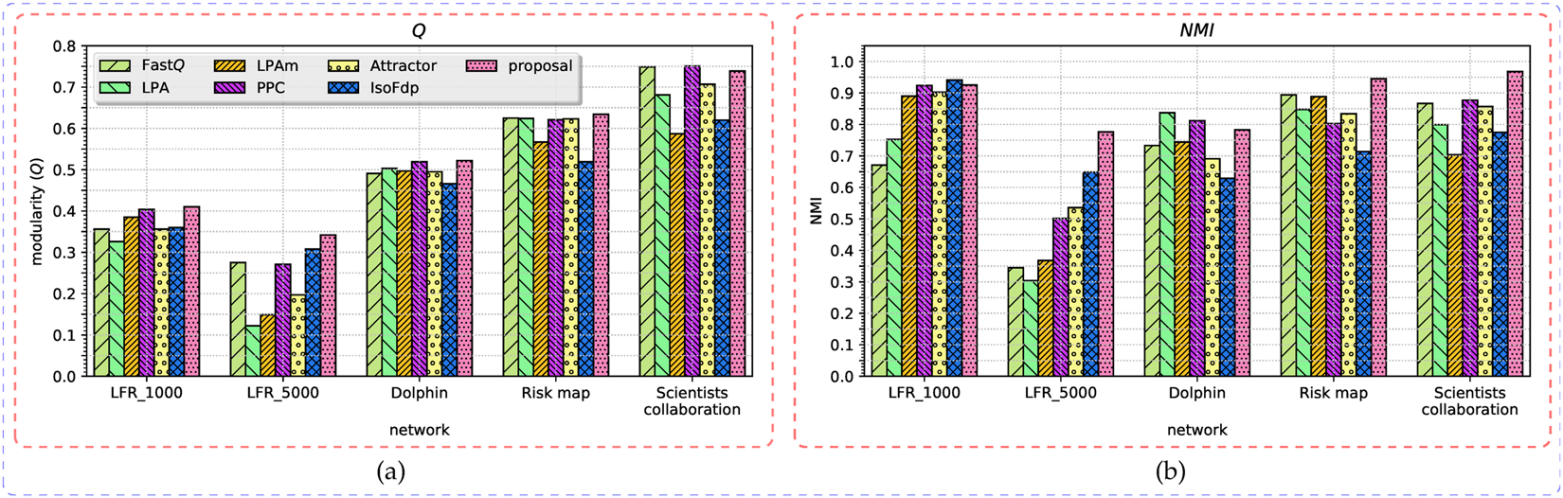
The metric values obtained from the first category of networks by the proposal and comparison algorithms. (**a**) The bar chart of the modularity (*Q*). (**b**) The bar chart of NMI.

**Figure 6:**
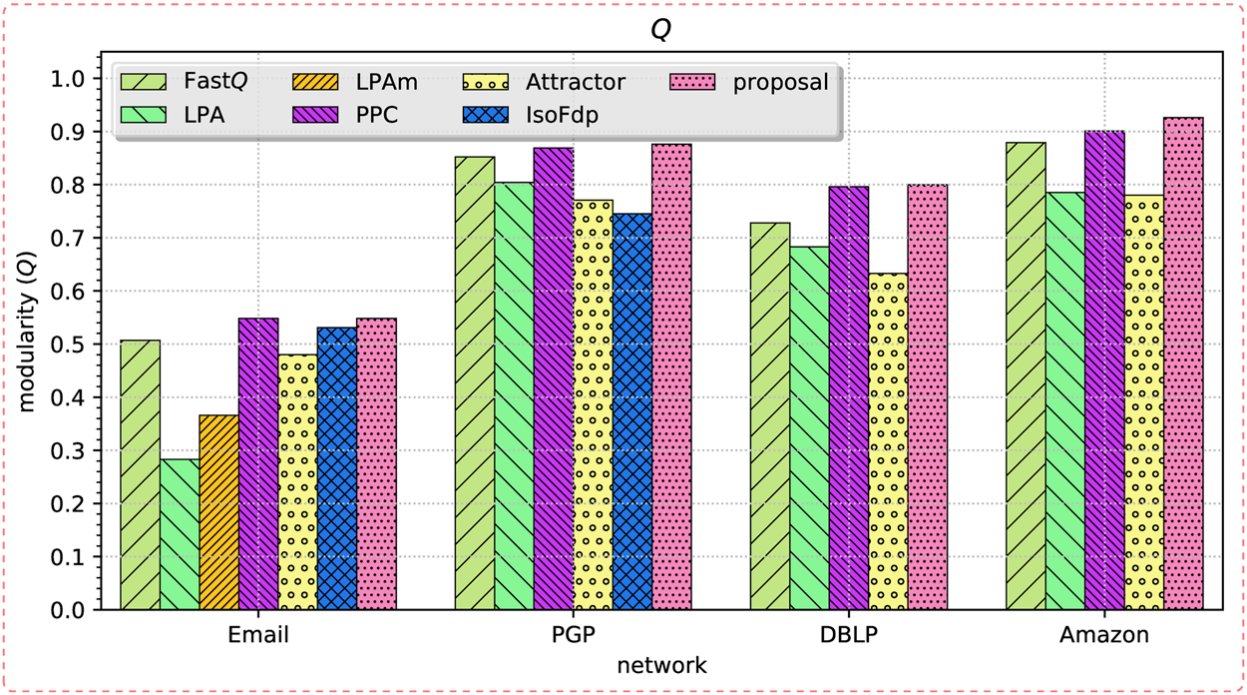
The bar chart of the modularity (*Q*) metrics obtained from the second category of networks.

For the first category of networks, we measure the quality of extracted community structures in terms of modularity (*Q*)^9^ and normalised mutual information (NMI)^47^. And for the second categories, we use the modularity as the measure only due to the absence of acknowledged ground-truth community structures. The comparison results on the two categories of networks are recorded in Tables 2 and 3, respectively. To illustrate the results more intuitively, we also plot the metric values as the bar charts in Figs 5 and 6, respectively.

**Table 3:** The experimental results on the second category of networks, the quality of the obtained results are measured using the modularity (*Q*). The largest value are typed in bold.

network	Fast*Q*	LPA	LPAm	PPC	Attractor	IsoFdp	proposal
Email	0.507	0.283	0.366	0.546	0.48	0.531	**0.547**
PGP	0.852	0.804	—	0.869	0.771	0.745	**0.878**
DBLP	0.728	0.683	—	0.796	0.633	—	**0.8**
Amazon	0.879	0.785	—	0.901	0.78	—	**0.926**

We can see that on the first category of networks, either *Q* or NMI or both of them obtained by the proposal are the largest. When measured using *Q*, our proposed method ranked the first on four of the networks, except on the scientists collaboration network only. When considering from the perspective of NMI, the proposed method achieved the largest value from three of the networks. On the other two networks, it still acquired the second and third largest NMI values, respectively. Other algorithms obtained better value of *Q* or NMI occasionally, i.e. *Q* of PPC from the scientists collaboration network, NMI’s of IsoFdp from the LFR_1000 network and LPA from the dolphin social network. On the second category of networks, all of the values of *Q* acquired by our method are the largest. PPC and Fast*Q* can also get the relative larger value of modularity from these networks, because they are both originated from modularity optimisation. LPAm and IsoFdp cannot manage to get a result from DBLP network and Amazon network, due to the larger size of the two networks; and LPAm cannot obtain a result even from the PGP network, which is only in ten-thousands-scale of vertices. It is mainly because that LPAm needs to calculate the modularity for each update of the vertex label in each iteration, which is a time-consuming work. For Attractor, it can get the definite results from all of these networks, but the obtained modularities are not so satisfactory. These results demonstrate that the proposed method can steadily extract high-quality community structures effectively from networks, and outperforms the comparison algorithms significantly.

## Discussions

The network involved in this paper is the undirected and unweighted graph, which can be denoted as *G* = (*V*, *E*), where *V* and *E* are vertex set and edge set, respectively.

The proposed method detects communities mainly from networks by simulating the voting behaviours in elections allowing nominating freely, the voting rules are of great importance to the detecting procedure. In the detecting procedure, each vertex $u\in V$ votes following the rules below:

1. If vertex *u* has been nominated as a candidate, or *u* has the largest degree among its neighbours, then *u* votes for itself.
2. Otherwise, we pick out the vertex whose degree is larger than that of *u* from the neighbours of *u* and denote it as *v*. If there are more than one such vertices, the one which is the most similar to *u* is selected and denoted as *v*. We denote the similarity between *u* and *v* as *sim*(*u*, *v*). If *sim*(*u*, *v*) = 0, then *u* nominates itself as a candidate and votes for itself.
3. Otherwise, if *v* has not voted for other vertices, then *u* nominates vertex *v* as a candidate and votes for *v*.
4. If vertex *v* has voted for another vertex *w*, that is to say *v* gives up its privilege being nominated as a candidate, then vertex *u* votes for *w* as well.

According to the above voting rules, the voting order of vertices can have influence on the order of vertices being nominated as candidates, so that different voting orders may lead to different results. In the presented method, we calculate the clustering coefficient for each vertex in the network, and have the vertices voted in the ascending order of their clustering coefficients. For any vertex $v\in V$, its clustering coefficient is defined as the ratio of the number of existed edges to the number of all possible edges in the neighbourhood of *v*, and can be calculated as

1 $$cc(v)=\frac{|\{(u,w)|u\in N(v),w\in N(v),(u,w)\in E\}|}{|N(v)|\cdot (|N(v)|-\mathrm{1)}},$$

where, $N(v)=\{u|(u,v)\in E,u\in V\}$ is the neighbour set of vertex *v*.

For a given vertex *v*, the larger the number of existed edges among its neighbours, the larger the clustering coefficient of *v*. If the sub-graph consisted of the neighbours of vertex *v* is a complete graph, *cc*(*v*) reaches its maximum, 1. However, it is unlikely that the neighbourhood sub-graph for any vertex in a sparse network is a complete graph, especially for vertices with larger degrees. In another word, the clustering coefficient for a vertex with larger degrees is always small. Therefore, voting in the ascending order of clustering coefficients of vertices can nominate the vertices with larger degrees as candidates earlier, then surround them with their neighbours to construct clusters.

According to the voting rule 2, if there are more than one vertex whose degree is larger than that of vertex *u* in the neighbourhood of vertex *u*, we use the similarity between those vertices and *u* to determine which one should be voted for by *u*. Therefore, the similarity between vertices plays an important role in the voting procedure as well. In the proposed method, we calculate the similarity between vertex *u* and *v* as follows,

2 $$sim(u,v)=\frac{|N(u)\cap N(v)|}{|N(u)\cup N(v)|-2}.$$

Because vertices *u* and *v* may be contained in $N(u)\cup N(v)$, but not in $N(u)\cap N(v)$, we subtract 2 in the denominator, so that *sim*(*u*, *v*) falls in the range of [0,1].

After voting, we can get a series of small clusters. However, these clusters are not the resulting communities. We know from the aforementioned observations that a final community can include several such clusters. Therefore, we take these clusters as initial communities, and consider to merge some of them to erect the resulting communities. In pursuit of high quality of the result, we only consider the pairs of communities whose similarity is larger than 0. In the experiments, we calculate the similarity between a pair of communities, *C*_*i*_ and *C*_*j*_, as follows,

3 $$SIM({C}_{i},{C}_{j})=\sum _{u\in {C}_{i}}\sum _{v\in {C}_{j}}sim(u,v\mathrm{)}.$$

## Methods

The proposed method consists of two phases in logical. The first phase is the voting procedure which yields several small clusters, and the second is the merging phase to take the small clusters as initial communities, and merge some of them into larger ones to erect the resulting community structure. The framework of the method is listed as Algorithm 1.

**Algorithm 1:**
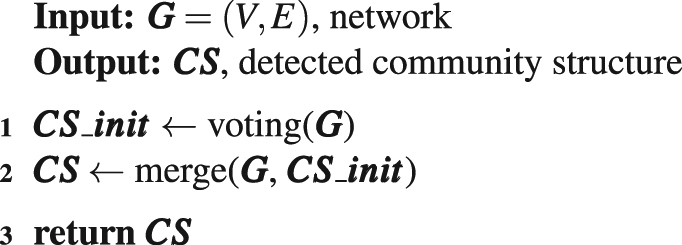
Voting Simulation based Agglomerative Hierarchical community detection method.

The function ‘voting()’ is corresponding to the voting procedure, in which each vertex in the network votes for the vertex with larger influential in its neighbourhood following the voting rules described in section ‘Discussions’, the logic is presented as the pseudo-code listed in Algorithm 2, and its output is some small clusters.

These small clusters are not the final communities, on the contrary, one community can be comprised of several such clusters. Therefore, we take the small clusters as initial communities, and function ‘merge()’ is responsible for merging some of the communities to construct the resulting community structure. In pursuit of efficiency, we borrowed the idea from Fast*Q*^15^ algorithm, and employed the similar strategy to join a pair of communities whose merge can lead to the largest modularity increment in each iteration when we implemented the function. According to ref.^15^, the increment of modularity by joining communities *C*_*i*_ and *C*_*j*_ is ${\rm{\Delta }}{Q}_{ij}=2({e}_{ij}-{a}_{i}{a}_{j})$, where *e*_*ij*_ is the proportion of inter-edges between *C*_*i*_ and *C*_*j*_ to total edges in the network, *a*_*i*_ and *a*_*j*_ are the ratio of edges incident to vertices located in *C*_*i*_ and *C*_*j*_ to total edges in the network, respectively, i.e., the modularity increment can be calculated quickly. The pseudo-code of the merge procedure is listed in Algorithm 3.

And this function outperforms Fast*Q* in some ways. This merge process gets started from the status that each small cluster is taken as an initial community, rather than from the status of each vertex being a community, which means that the joining times needed here is far less than that of Fast*Q*. In addition, because merging the dissimilar communities may undermine the quality of resulting community structure, we add the consideration of only merging the two communities whose similarity is larger than 0 in each iteration, and the calculation of similarity between two communities is defined as equation (3) in section ‘Discussions’. And moreover, we can terminate the joining procedure earlier when there is no similarity between any pair of communities larger than 0, rather than repeat the joining operation until all vertices are in the same community. In this way, the merge process here works with a high efficiency.

**Algorithm 2:**
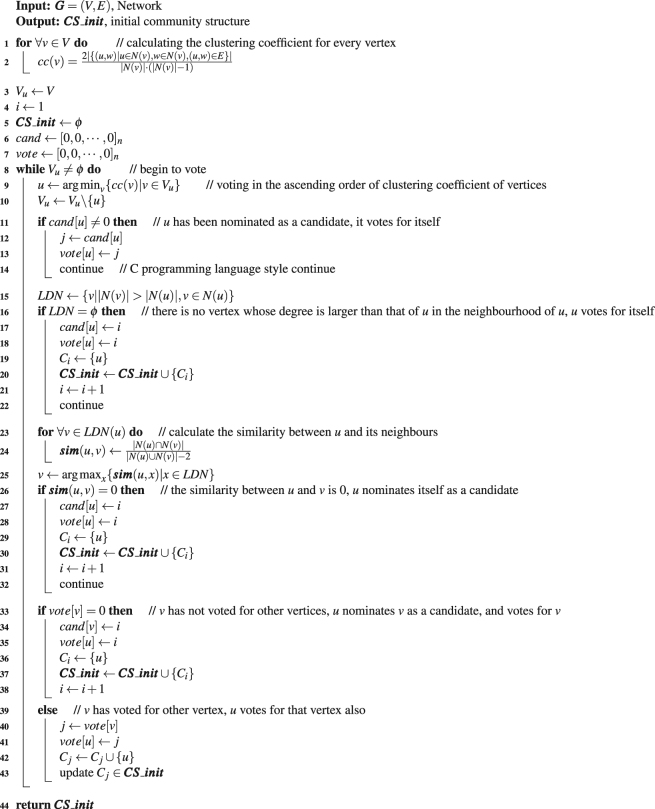
The logic of function voting (***G***).

**Algorithm 3:**
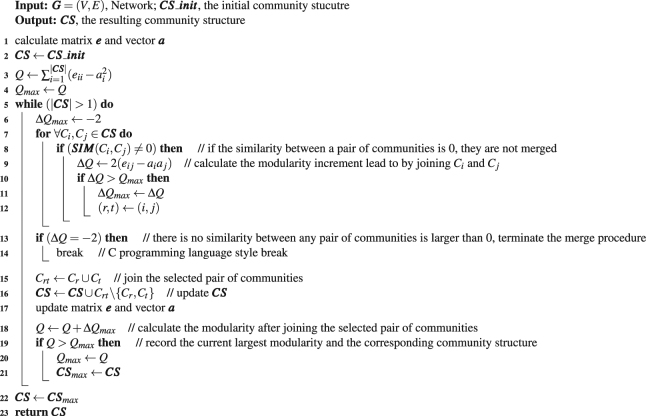
The logic of function merge (***G***, ***CS***_***init***).

### Complexity Analysis

For an algorithm, it is advantageous if it has a lower time complexity, so that it can be applied to large-scale networks, which are ubiquitous in the big-data era, now. Our proposed method consists of two phases, the first phase is to obtain the initial communities by simulating the voting procedure. This task can be accomplished in $O(m\bar{d}+n\,\mathrm{log}\,n)$, where *n* and *m* are the number of vertices and edges in the network, respectively; $\bar{d}$ is the average degree of vertices. The second phase is to acquire the final communities by merging some of the initial communities, and has a cost time complexity *O*(*mk*), where *k* is the merging-iteration times, and $k\ll n$, $\bar{d} < k$ in general cases. Therefore, the total time consumption of our method is $O(m\bar{d}+n\,\mathrm{log}\,n+mk)\sim O(mk)$, or *O*(*nk*) in sparse networks. As a result, the proposed method can be efficiently applied to large-scale networks, which can be manifested to some extent by the experimental results.
